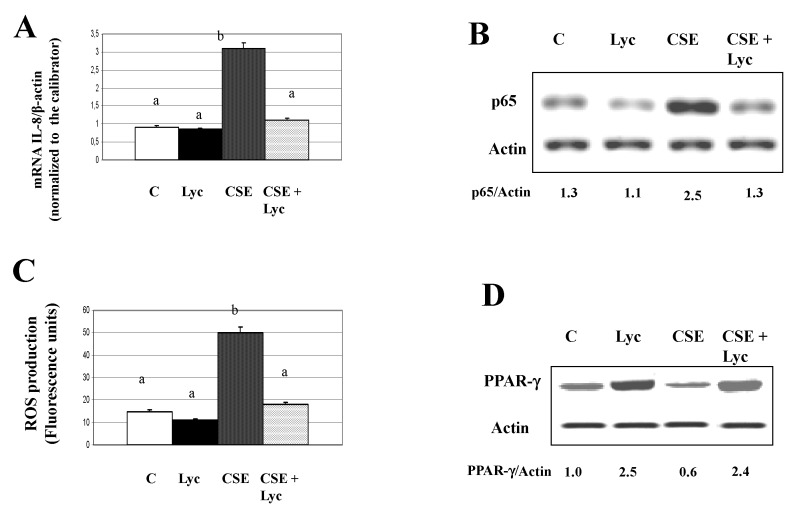# Correction: Lycopene Inhibits NF-kB-Mediated IL-8 Expression and Changes Redox and PPARγ Signalling in Cigarette Smoke–Stimulated Macrophages

**DOI:** 10.1371/annotation/4993e0e2-c580-4547-90d8-3227b87e6ae9

**Published:** 2013-02-28

**Authors:** Rossella E. Simone, Marco Russo, Assunta Catalano, Giovanni Monego, Kati Froehlich, Volker Boehm, Paola Palozza

Several of the figures in the published article had been generated using bands or after carrying adjustments to the original blots. The authors are issuing this Correction to provide the following corrected figures:

Figure 1: Duplicate bands from the original blot were cut to generate the figure. We report the entire blot for IL-8 at 6 h and at 24 h, indicating the different concentrations of CSE in duplicate. Since the bands increased, two panels have been added instead of one (Panel A: 6 h and panel B: 24 h). The revised figure legend is as follows:

'Figure 1. Effects of Cigarette smoke extract (CSE), alone and in combination with lycopene, on IL-8 production in human THP-1 cells. Panels A-E: intracellular IL-8 production; panel F: IL-8 production in culture medium. Panel A: effects of different CSE concentrations for 6 h, panel B: effects of different CSE concentrations for 24 h; panels C, D, E: effects of a pre- treatment for 6 h with lycopene (2 mM) followed by a 24-h CSE (0.5%) exposure; panel D: effects of a pre-treatment with different concentrations of lycopene followed by a 24-h CSE (0.5%) exposure; Panels A, B, C, D: representative Western Blot analyses; the values indicated represented the ratio of IL-8 and actin. Panel E: mRNA levels by reverse transcription polymerase chain reaction.

Panel F: Chemiluminescence Immunometric Assay. In the different panels, the values were the means ± SEM of three independent experiments. Values not sharing the same letter were significantly different (P<0.05, Fisher's test).'

Figure 2D. In the published figure, the bands for the different raw blots (p-IKKa, p-IKba, IKba and actin) had been cut and pasted to align the bands in the figure, we attach the original blot.

Figures 2C, 3C, 5B, 5D and 6D. The published figures were processed to eliminate background, we report the original blots.

Figure 5C. In the published figure the actin bands were rotated, we present the corrected figure. 

Updated Versions of Figures:

**Figure:**
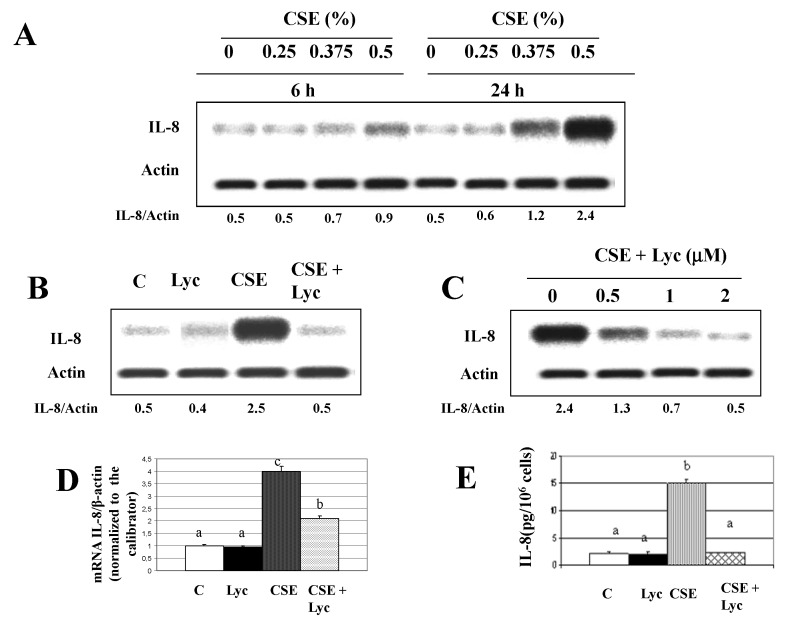


**Figure:**
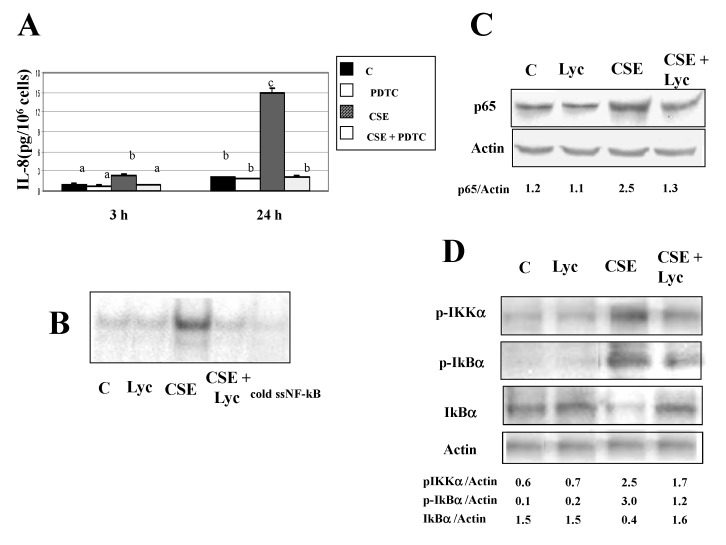


**Figure:**
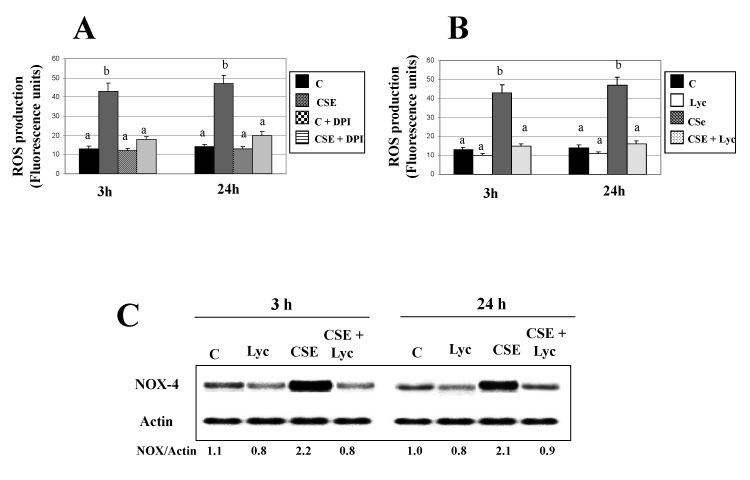


**Figure:**
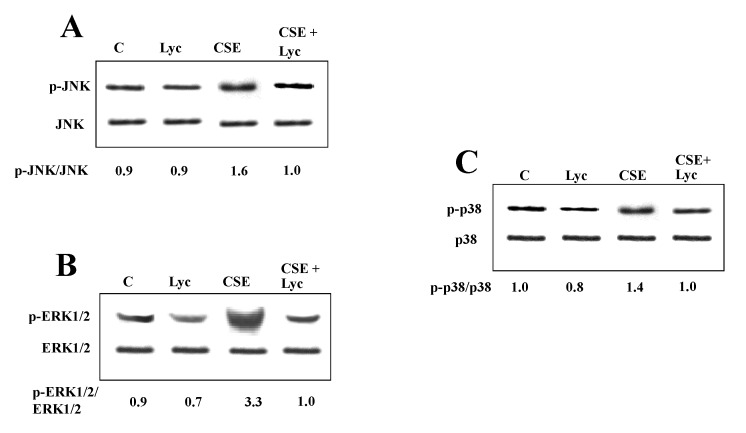


**Figure:**
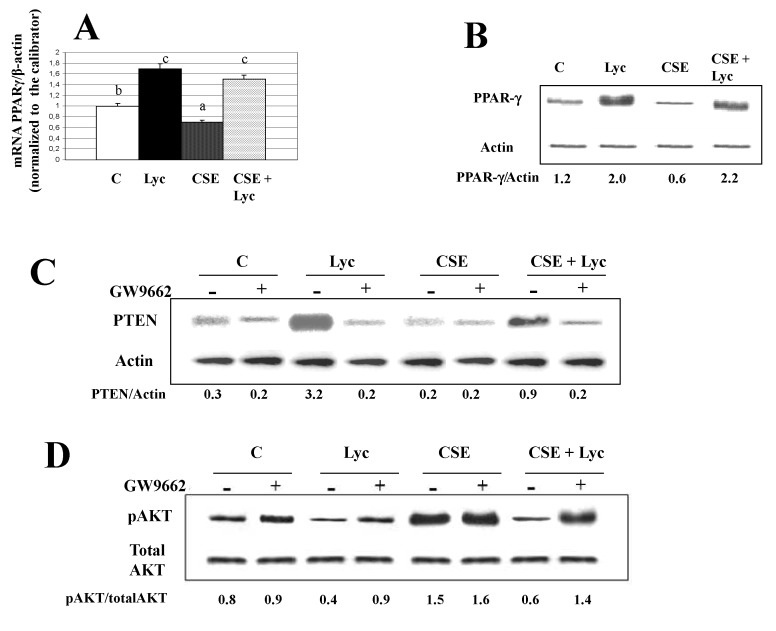


**Figure:**